# Reuse of N95/PFF2 masks in clinical practice: morphological and structural analysis ^*^

**DOI:** 10.1590/1518-8345.7045.4209

**Published:** 2024-07-05

**Authors:** Viviane Lopes Vimieiro, Claysson Bruno Santos Vimieiro, Adriana Cristina de Oliveira

**Affiliations:** ^1^ Universidade Federal de Minas Gerais, Escola de Enfermagem, Belo Horizonte, MG, Brazil.; ^2^ Scholarship holder at the Fundação de Amparo à Pesquisa do Estado de Minas Gerais (FAPEMIG), Brazil.; ^3^ Pontifícia Universidade Católica de Minas Gerais, Belo Horizonte, MG, Brazil.; ^4^ Universidade Federal de Minas Gerais, Belo Horizonte, MG, Brazil.

**Keywords:** Personal Protective Equipment, N95 Respirators, Equipment Reuse, Health Personnel, Occupational Health, Microscopy Electron Scanning

## Abstract

**Objective:**

to analyze the integrity of N95/PFF2 masks in relation to fiber morphology, porosity, cracks and micro holes, as well as identify visible damage to their structure and components, after seven- and fifteen-day reuse protocols.

**Method:**

cross-sectional study. Structural and morphological characteristics of a new N95/PFF2 mask were analyzed in comparison with N95/PFF2 masks (n=10) used in seven- and fifteen-day protocols, through visual inspection and scanning electron microscopy.

**Results:**

upon visual inspection, following the seven-day protocol, 40% and 60% of the N95/PFF2 masks showed, respectively, personal identification marks and external and internal dirt. Additionally, 20% exhibited loosening and/or tearing of the straps, while 100% showed some type of damage to the nose clips. In the fifteen-day protocol, all N95/PFF2 masks had dirt, loose straps and damaged nose clips, and 80% had folds. Electronic microscopy revealed an increase in pores and loosening in the weaves from seven days onwards, extending up to fifteen days, with the presence of micro holes and residues.

**Conclusion:**

the reuse of N95/PFF2 masks affects their structural and morphological integrity. It is crucial to carry out tests to measure the impact of this practice on the safety of health professionals.

## Highlights

**(1)** Morphological and structural alterations in N95/PFF2 masks after reuse.

**(2)** Impact on the integrity and functionality of N95/PFF2 masks after reuse.

**(3)** Implications for the safety of health professionals.

**(4)** Need to reevaluate reuse protocols.

**(5)** Scanning Electron Microscopy as a valuable tool.

## Introduction

 The N95/PFF2 mask is the main Personal Protective Equipment (PPE) used in the prevention and control of respiratory diseases such as tuberculosis, measles, COVID-19 and H5N1 influenza ^(^ ^1^ ^)^ . The structural integrity of N95/PFF2 masks is essential to ensure facial fit and a minimum filtration efficiency of 95% of airborne particles up to 0.3 microns (µm), in order to ensure the safety of health professionals ^(^ ^2^ ^)^ . 

 The Occupational Safety and Health Administration (OSHA) requires the N95/PFF2 mask to be used only once and discarded immediately after use ^(^ ^3^ ^)^ . However, shortages of this equipment during epidemics, such as H1N1 influenza in 2009 and the Middle East Respiratory Syndrome in 2012, and the COVID-19 pandemic, have led bodies such as the Centers for Disease Control and Prevention (CDC), the *Agência Nacional de Vigilância Sanitária* (ANVISA) and other health organizations recommending the implementation of limited reuse protocols ^(^ ^4^ ^-^ ^6^ ^)^ . These protocols include the use of N95/PFF2 masks in multiple appointments or work shifts, and their removal and storage for subsequent use ^(^ ^5^ ^)^ . 

 Despite being justifiable due to the scarcity of this device, such recommendations may compromise the structural integrity of N95/PFF2 masks, resulting in possible harm to the respiratory protection offered to users. In fact, studies conducted among professionals working in emergency services revealed that N95/PFF2 masks reused for more than three eight-hour shifts showed considerably high failure rates (≥ 40%) in sealing tests, a phenomenon possibly attributed to the inadequate fit of the PPE to the face ^(^ ^7^ ^-^ ^8^ ^)^ . 

 Additional risks associated with reuse protocols include the need, inherent to clinical practice, for health professionals to take breaks to use the bathroom, have meals, and even rest their face. These circumstances imply a greater number of donning and doffing, which can lead to the deterioration of the components of the N95/PFF2 masks, such as loosening or breaking of the straps, breakage of the nose clip, folds, cracks and tears ^(^ ^9^ ^-^ ^10^ ^)^ . 

 N95/PFF2 masks are often made up of four distinct layers: two intermediate layers responsible for filtration and support, and outer and inner layers that do not filter particles ^(^ ^11^ ^-^ ^12^ ^)^ . The filtration effectiveness of an N95/PFF2 mask is directly related to the preservation of the morphological characteristics of the fibers in each layer. However, degradation of filter material may not be detected by health professionals, resulting in a potential reduction in respiratory protection ^(^ ^11^ ^)^ . 

 Previous studies on the impacts of reusing N95/PFF2 masks were mainly based on testing the seal and performing visual inspection ^(^ ^8^ ^-^ ^10^ ^,^ ^13^ ^-^ ^15^ ^)^ . Nevertheless, these methods may be insufficient when seeking an objective assessment of the effectiveness of N95/PFF2 masks. Furthermore, the lack of clear guidelines regarding maximum reuse time leaves a gap in knowledge. 

 To address this gap, this study adopts a comprehensive strategy that combines traditional visual inspection with advanced technique such as scanning electron microscopy (SEM). This approach allows for a more detailed and precise analysis, capable of identifying microscopic morphological damage that may not be visible to the naked eye ^(^ ^16^ ^-^ ^17^ ^)^ . 

Due to the scarcity of evidence on the safe reuse time of N95/PFF2 masks that ensures the preservation of structural integrity, this study aims to analyze the integrity of N95/PFF2 masks in relation to fiber morphology, porosity, cracks and micro holes, as well as identifying visible damage to its structure and components after seven- and fifteen-day reuse protocols.

## Method

### Study design and scenario

 Cross-sectional study. The scenario included two Intensive Care Units (ICU) located in two public hospitals in Belo Horizonte, MG, Brazil, with a capacity of 10 and 54 beds, respectively. The choice of these institutions was based on the difference in the reuse protocols for N95/PFF2 masks adopted, seven days for the first and fifteen days for the second. Each unit had exclusive, airy and ventilated spaces intended for the safe storage of these devices. The reuse protocols were drawn up by the *Setor de Segurança do Trabalho* (SST) and the *Comissão de Controle de Infecção Hospitalar* (CCIH) of each hospital, with detailed guidance for health professionals on correct packaging, highlighting the importance of using appropriate paper and clear identification of each N95/PFF2 mask with user name and time of use. 

### Population

The eligible population consisted of nursing professionals working in direct care in the selected units.

### Selection criteria

N95/PFF2 masks used by nursing professionals during the day shift were included, in accordance with reuse protocols (seven and fifteen days). The choice of this shift was due to the greater number of procedures performed during this period. N95/PFF2 masks used by professionals who did not follow the time intervals determined by the reuse protocols were excluded.

### Sample definition

 The convenience sample was divided into two comparison groups: one group with five N95/PFF2 masks used for seven days, and the other with five used for fifteen days, both following the established reuse protocol. It is important to highlight that all N95/PFF2 masks were of the same brand and model, with one standard size. This particular model is characterized by its two elastic material straps, uncoated metal nose clip for personalized fit and foldable design. Furthermore, all devices had a Certificate of Approval (CA) issued by the *Ministério do Trabalho e Emprego* (MTE), ensuring that they met the rigorous safety and quality standards required by Brazilian legislation. 

### Study variables

 The study variables were analyzed using two data collection instruments, both developed by the researchers and based on the regulations of the US Occupational Safety and Health Administration and the Brazilian *Ministério do Trabalho e Emprego* on the safe use of the N95/PFF2 mask. The first instrument evaluated the structural integrity of N95/PFF2 masks, considering the time of use (seven or fifteen days), the professional category (nurses or nursing technicians), and the presence of personal identification marks, folds, holes or tears on the surface, stains and dirt on the external and internal parts (with yes or no answers for each item). In affirmative cases, the types of stains or dirt were specified, including traces of makeup, yellowish or blackened stains, coffee, pen scribbles, among others. To quantify the extent of dirt or stains, an estimate in multiples of 25% of the compromised area of the N95/PFF2 mask was adopted. This approach included a visual assessment, followed by classification of affected areas into categories according to their extent. Additionally, the conservation status of the straps (intact or stretched) and the nose clip (intact, crushed or creased, broken, scored, or other specified conditions) was evaluated. 

The second instrument focused on the morphological characteristics of the fibers of the different layers of N95/PFF2 masks, including the time of use (seven or fifteen days), the professional category (nurses or nursing technicians), the specific layers (external, structural, filtering and internal), the characteristics of the fibers (well preserved, irregular or highly irregular), the presence of imperfections (micro holes, cracks or both) and residues. The region chosen for sample collection was the area close to the nasal part, on the right side of the N95/PFF2 mask, selected due to the greater degree of humidity caused by the user’s breathing.

### Period, instruments used to collect information and data collection

Data collection was carried out between February and April 2023. Daily, at the end of each day shift, N95/PFF2 masks were collected according to a schedule previously established together with the health services. These samples were packed in brown envelopes, labeled with information about the place and date of collection, device brand, period of use and the user’s professional category. Subsequently, they were stored in rigid boxes that had been cleaned with soap and water, in a ventilated environment. During the process, safety measures were strictly adopted, including the use of aprons, procedure gloves and surgical masks.

 The researcher was responsible for transporting the N95/PFF2 masks, already packed in boxes, from the intensive care units to the Engineering Center of the *Pontifícia Universidade Católica de Minas Gerais* (PUC Minas), in Belo Horizonte, Brazil. At the Center, structural integrity was analyzed through visual inspection in natural light. An optical lens with ten times magnification (Tomshin S.A., São Paulo, SP, Brazil) was used on a benchtop disinfected with 70% alcohol to detect the presence of identification marks, folds, holes or tears, stains and dirt, and also to assess the state of conservation of the components of the N95/PFF2 mask. Identification marks indicated markings made by the user, usually with a ballpoint pen, to distinguish their device and control usage time. The results were recorded on individual forms for each sample. 

 After collection and visual inspection, all N95/PFF2 masks were sent to the Advanced Microscopy Laboratory at *PUC Minas* . In this laboratory, a scanning electron microscope (JSM-6610LV, Jeol, Tokyo, Japan) was used to analyze the morphology of the fibers in the different layers of the device. Manipulation was performed with non-sterile gloves and apron on a benchtop. For analysis, the samples were sectioned close to the nose region, on the right side, using a scissor previously disinfected with 70% alcohol. The sections had approximate dimensions of 1.0 x 1.0 cm. Then, the layers were separated and fixed directly to an aluminum “stub” with double-sided carbon adhesive tape. To make the surfaces conductive for SEM visualization, a fifteen-nanometer-thick gold film was deposited on the samples using a high-pressure vacuum coating system (Denton Vacuum Desk S.A, New Jersey, United States of America). The micrographs were obtained at a magnification of 100x, 500x and 1000x. It is worth noting that, shortly after arriving at the Engineering Center and the Microscopy Laboratory, the N95/PFF2 masks were immediately subjected to testing. This procedure was adopted to prevent the influence of any environmental variables resulting from storage in inappropriate locations on the results. 

### Data processing and analysis

Use of the Statistical Package for the Social Sciences (SPSS), version 28.0, for descriptive analyzes and presentation of data in absolute and relative frequency distribution tables.

### Ethical aspects

The study followed the ethical principles of Resolution 466/2012 of the National Health Council, referring to research involving human beings, receiving approval from the Research Ethics Committee (CEP, for its acronym in Portuguese) of Brazil, under opinion number: 5,824,571 - Certificate of Presentation of Ethical Assessment (CAAE, for its acronym in Portuguese): 65232922.8.0000.5149. Nursing professionals were invited to participate on a voluntary basis, and all pertinent information was provided, ensuring the individual signature of the Free and Informed Consent Form (TCLE, for its acronym in Portuguese). In order to preserve anonymity, the data collection process was designed to exclude any data that could identify the participating individuals.

## Results

**Figure 1:**
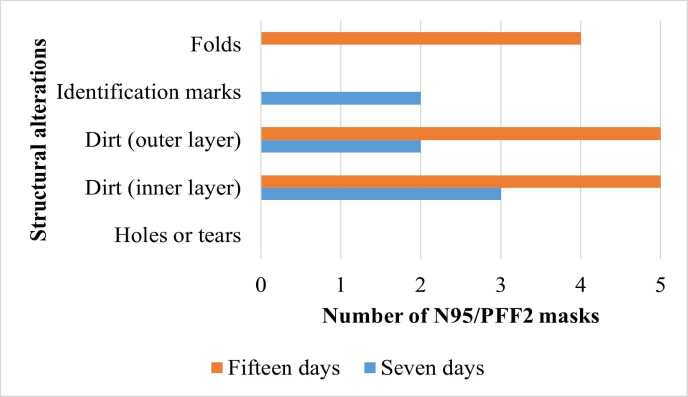
- Structural alterations in N95/PFF2 masks after use in seven (n=5) and fifteen-day (n=5) protocols, on a 12-hour shift. Belo Horizonte, MG, Brazil, 2023

 The integrity of the N95/PFF2 masks in relation to the reuse protocol (seven and fifteen days) was evaluated through visual inspection with the aid of an optical lens with ten times magnification from the Tomshin ^®^ brand ( Figure 1 ). 

Regarding the structure of the N95/PFF2 masks, the presence of identification marks was observed in 40% (2/5) of the devices that were reused for seven days. These marks, made by nursing technicians, consisted of writing the initials of their names and the date of receipt, using ballpoint pens on the outside of the N95/PFF2 masks. The purpose of these markings was to identify and control the usage time of each piece of equipment.

With regard to dirt, after seven days of use, 40% (2/5) of the N95/PFF2 masks had yellowish or blackened stains of undetermined origin on at least 25% of their surface in the outer layer, while 60% (3/5) had dirt on the inner layer. As for the samples used for fifteen days, all of them showed dirt on both layers, with the stains occupying an area of less than 25% on the outer layer, and on the inner layer the majority, 60% (3/5), showed traces of makeup in an area greater than 75%.

Regarding the components of N95/PFF2 masks reused for seven days, 20% (1/5) of the straps showed loosening and 20% (1/5) broke; 100% (n=5) of the nose clips had some kind of damage: 20% (1/5) were dented or had fold marks, 20% (1/5), in addition to this damage, also had grooves, and 60% (3/5) had only grooves. In all N95/PFF2 masks reused for fifteen days there was loosening of the straps and compromise of the nose clip, with dents or fold marks and grooves. Furthermore, in 40% (2/5) of the samples from the fifteen-day protocol, defoliation was observed between the layers on the inside and the presence of foam dressings inserted by health professionals for nasal protection.

All N95/PFF2 masks were subjected to scanning electron microscopy to analyze the morphological alterations in the fibers of each layer, after being used in reuse protocols. This analysis revealed that all samples showed similar morphological modifications. Given this consistency in findings, an N95/PFF2 mask representative of each protocol was selected for illustration in the study figures. In comparison, the control (unused) N95/PFF2 mask displayed in the micrographs a well-preserved fiber contour surface, without micro holes, cracks or adhered residues.

**Figure 2:**
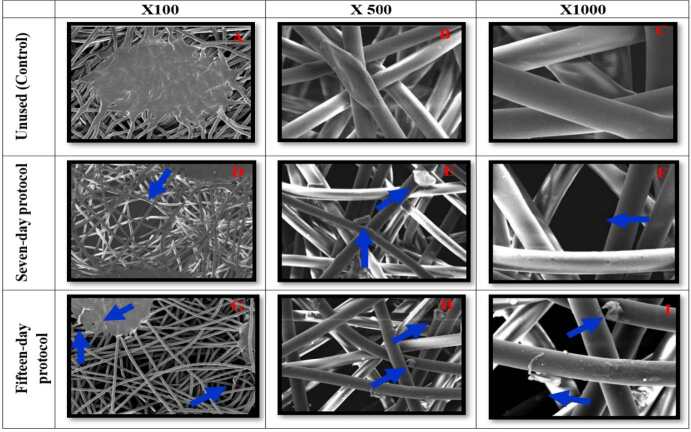
- Scanning electron microscopy of the outer layer of the control N95/PFF2 mask and of the samples in the reuse protocols (seven and fifteen days) during 12-hour shifts, with magnification of 100x, 500x and 1000x. Belo Horizonte, MG, Brazil, 2023

 When examining the outer layers of the seven-day samples, the presence of pores between the weaves and residues adhered to the fibers was observed. In the fifteen-day samples, micro holes, loose fiber strands were observed, as well as residues adhered to the fibers and pores between the weaves ( Figure 2 ). 

Microscopic images 2(A-C) of the outer layer of the control N95/PFF2 mask revealed well-preserved fibers. In images 2(D-F), referring to seven-day samples, the presence of pores between the weaves in 2(D) and 2(F) and residues in 2(E) can be observed. In images 2(G-I), corresponding to fifteen-day samples, micro holes and loose fibers were identified in 2(G), residues in 2(H) and 2(I), and pores between the weaves in 2(I).

**Figure 3:**
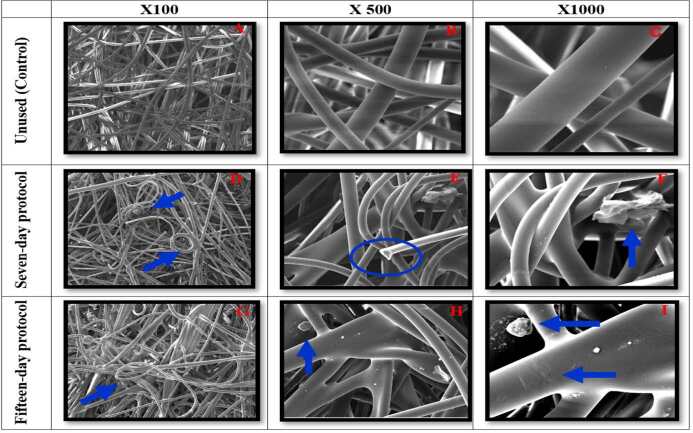
- Scanning electron microscopy of the structural layer of the control N95/PFF2 mask and of the samples in the reuse protocols (seven and fifteen days) during 12-hour shifts, with magnification of 100x, 500x and 1000x. Belo Horizonte, MG, Brazil, 2023

 In the analysis of the structural layers of the N95/PFF2 masks after the seven-day reuse protocols, “entangled” weaves, fiber breakage and residues adhered to them were observed. In the samples reused for fifteen days, in addition to the “entangled” weaves and adhered residues, wear on the fibers was also identified ( Figure 3 ). 

**Figure 4:**
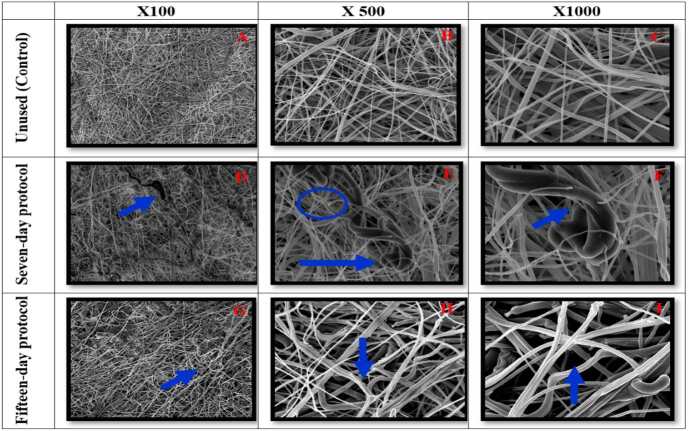
- Scanning electron microscopy of the filtering layer of the control N95/PFF2 mask and of the samples in the reuse protocols (seven and fifteen days) during 12-hour shifts, with magnification of 100x, 500x and 1000x. Belo Horizonte, MG, Brazil, 2023

 Microscopic images 3(A-C) of the control N95/PFF2 mask showed well-preserved fibers. Image 3(D) of the seven-day samples revealed “entangled” weaves, image 3(E) showed fiber breakage and residues, while image 3(F) showed only residues. Images 3(G-I) of the fifteen-day samples revealed “entangled” weaves in 3(G), residues in 3(H) and residues with fiber wear in 3(I). Regarding the filtering layers of the N95/PFF2 masks subjected to the seven- and fifteen-day reuse protocols, the presence of “entangled” weaves and fiber breakage was observed. In fifteen-day samples, in addition to “entangled” weaves, larger pores were found between the weaves ( Figure 4 ). 

Images 4(A-C) of the control N95/PFF2 mask showed well-preserved fibers. Images 4(D-F) of the seven-day samples revealed “entangled” weaves. Images 4(G-H) of the fifteen-day samples also revealed “entangled” weaves, while image 4(I) revealed pores between the weaves.

**Figure 5:**
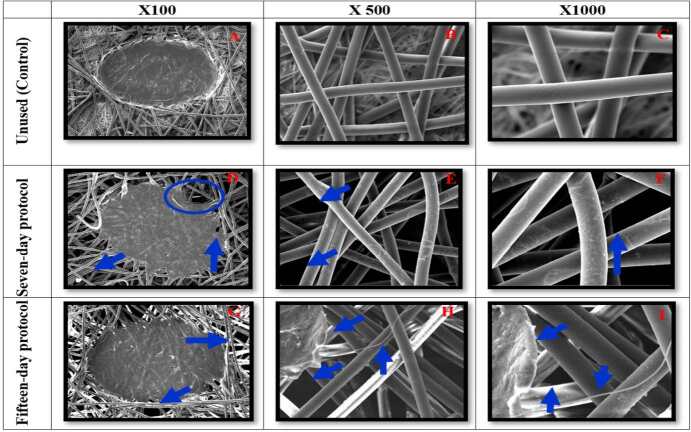
- Scanning electron microscopy of the inner layer of the control N95/PFF2 mask and of the samples in the reuse protocols (seven and fifteen days) during 12-hour shifts, with magnification of 100x, 500x and 1000x. Belo Horizonte, MG, Brazil, 2023

 When analyzing the internal layers of the N95/PFF2 masks used in the seven-day protocols, micro holes, residues adhered to the fibers and pores between the weaves were identified. In the samples reused in the fifteen-day protocols, in addition to this damage, the presence of loose fiber strands was revealed ( Figure 5 ). 

Images 5(A-C) of the control N95/PFF2 mask showed well-preserved fibers. Image 5(D) of the seven-day samples revealed the presence of micro holes, residues and pores between the weaves, while images 5(E) and 5(F) revealed pores between the weaves. Image 5(G) of the fifteen-day samples also revealed pores between the weaves, and images 5(H) and 5(I) showed residues and pores between the weaves, as well as loose fiber strands.

## Discussion

In the analysis of N95/PFF2 masks subjected to reuse protocols for periods of seven and fifteen days, substantial alterations were found attributable to this practice, affecting both the structural integrity and the morphology of the fibers. This fact suggests an increased risk to the safety of health professionals, highlighting the need for a cautious and judicious approach when reusing this protective equipment.

 These findings are of great relevance, given that the implementation of these reuse protocols was essential to meet the high global demand during the COVID-19 pandemic, as well as in previous epidemics, such as H1N1 influenza in 2009 and Middle East Respiratory Syndrome in 2012 ^(^ ^18^ ^-^ ^21^ ^)^ . Furthermore, this strategy is still adopted as a policy to reduce financial costs in several hospital institutions around the world, especially in countries that face disproportionate impacts from COVID-19, such as Brazil, India and China, with high numbers of cases and mortality, in addition to a high incidence of other respiratory diseases, such as tuberculosis ^(^ ^22^ ^-^ ^24^ ^)^ . 

 When evaluating the integrity of the N95/PFF2 masks reused for seven and fifteen days, several signs of wear on the straps and degradation on the nose clips were identified through visual inspection. These damages corroborate the findings of studies carried out in both clinical and laboratory environments, highlighting the fragility of this equipment under reuse conditions ^(^ ^9^ ^,^ ^25^ ^)^ . A clinical study, carried out in a tertiary hospital in India during the COVID-19 pandemic, indicated that 9% of health professionals reported the loosening of straps, generally after five reuses in eight-hour shifts, as one of the main reasons for early disposal of N95/PFF2 masks ^(^ ^25^ ^)^ . In a laboratory context, simulating a ten-hour shift, it was found that the rupture of five nose clips and eight fixation items, which occurred over 20 episodes of donning and doffing, resulted in the failure of sealing tests for all participants who used such devices ^(^ ^9^ ^)^ . 

 The degradation of the components of N95/PFF2 masks can be exacerbated by the intense routine of health professionals, who often need to don and doff several times during the workday, especially for essential breaks such as hydration, eating, and even face rest ^(^ ^9^ ^,^ ^15^ ^)^ . Researches carried out in health services show a variation in the frequency of donning, ranging from up to five occurrences in ten-hour shifts and an average of forty in twelve-hour shifts ^(^ ^26^ ^-^ ^27^ ^)^ . Therefore, it is vital that users carry out rigorous visual inspections and check the seal of N95/PFF2 masks before each use to ensure the integrity of these devices is maintained and, consequently, ensure effective protection against airborne infectious agents ^(^ ^4^ ^-^ ^5^ ^,^ ^28^ ^)^ . However, a recent study revealed that 57% of nurses do not carry out these checks, highlighting the urgent need for frequent training and greater awareness about the essentiality of these procedures for the correct and safe use of personal protective equipment ^(^ ^4^ ^,^ ^29^ ^)^ . 

 Another critical point identified in the present study was the alarming incidence of dirt on N95/PFF2 masks, a reality that goes against health guidelines, which recommend the immediate replacement of devices that are dirty, humid or damaged ^(^ ^2^ ^,^ ^4^ ^)^ . This observation is corroborated by research carried out in a hospital environment, in which visual inspection revealed the presence of stains or dirt on all N95/PFF2 masks used by nursing assistants after five reuses, with cosmetic residues being the most common ^(^ ^10^ ^)^ . These findings highlight the critical need to adhere to practices recommended by health agencies, both in terms of inspection and maintenance of equipment free from contamination and dirt, as well as intact to ensure the effective protection of health professionals. Nevertheless, it should be noted that the use of makeup intensifies the deterioration of fibers, which, in turn, leads to a decrease in their effectiveness ^(^ ^4^ ^-^ ^5^ ^)^ . 

 The hospital institutions participating in the study, in accordance with the guidelines established by the health bodies ANVISA and CDC, provided their own, clean and ventilated places to store the N95/PFF2 masks ^(^ ^2^ ^,^ ^4^ ^)^ . Furthermore, they promoted the use of paper envelopes, which facilitate air circulation and allow personal identification, avoiding direct markings on the devices. These practices aim to preserve the structural integrity of the PPE and reduce the risk of inadvertent changes between users. However, after the seven- and fifteen-day protocols, folds and marks made by a professional with ballpoint pens were observed in the samples, suggesting possible improper packaging of the N95/PFF2 masks in clothing pockets or in personal items, such as bags. This condition is also observed in other studies, drawing attention to the cruciality of implementing awareness actions to discourage inappropriate storage of these devices ^(^ ^25^ ^,^ ^27^ ^)^ . 

 Another aspect that deserves to be highlighted in the context of the N95/PFF2 mask reuse protocol is the occurrence of adverse events, including pressure injuries in the nasal bridge due to friction and shear caused by the displacement of the device ^(^ ^30^ ^-^ ^31^ ^)^ . In clinical practice, it is common for health professionals to use silicone or foam adhesives on the inside of N95/PFF2 masks to protect the skin against damage and provide comfort in the region, especially the nose, due to the metal structure of the nose clip ^(^ ^32^ ^-^ ^33^ ^)^ . These strategies were observed in two samples reused in fifteen-day protocols in this study. However, these foam dressings can compromise the fit of these devices, due to the possibility of gaps between the skin and the respiratory protective equipment ^(^ ^32^ ^)^ . 

 The N95/PFF2 masks, certified by the National Institute for Occupational Safety and Health (NIOSH), are made of synthetic polypropylene microfibers, distributed in four distinct layers: external, structural, filtering and internal ^(^ ^28^ ^,^ ^34^ ^)^ . The outer and inner layers are specifically designed to protect against humidity, with fiber diameters between 20 and 25 microns ^(^ ^34^ ^)^ . On the other hand, the filtering medium is made up of smaller fibers, from two to five microns, electrostatically treated to retain at least 95% of airborne particles up to 0.3 µm ^(^ ^34^ ^)^ . 

From the seventh day of using the N95/PFF2 masks, scanning electron microscopy analyzes revealed a tendency for pores to enlarge in most layers and the presence of “entangled” weaves in the structural and filtering layers, possibly resulting from the detachment of the fibers, creating larger spaces between them. These alterations were intensified in the fifteen-day protocol of use, highlighting the direct relation between reuse time and the magnitude of the results observed.

 In the context of occupational health, these morphological alterations in device fibers raise concerns, as they substantially increase the possibility of blood, respiratory or nasal secretions and other bodily fluids penetrating through the layers of N95/PFF2 masks. This scenario is especially critical in environments such as intensive care units, where procedures that generate aerosols are routine and present a high risk of transmitting respiratory viruses ^(^ ^35^ ^-^ ^36^ ^)^ . 

 Furthermore, an additional study emphasizes that N95/PFF2 masks with smaller pores, closer to their original characteristics, offer superior filtration efficiency ^(^ ^37^ ^)^ . This means that greater spaces between the weaves favor the penetration of a greater number of aerosolized particles, increasing the vulnerability of these professionals ^(^ ^37^ ^-^ ^38^ ^)^ . 

Micrographs of the N95/PFF2 masks also showed the presence of micro holes and the rupture of the fibers, possibly resulting from fraying, which leads to the expansion of particle retention spaces and facilitates the accumulation of residues between the fibers. Elements observed during visual inspection, such as dirt, folds and personal identification marks on the samples, may have influenced these morphological alterations. Therefore, it is crucial to establish adequate protocols and limits for reuse of these devices to ensure the necessary protection for health professionals.

This study, despite its valuable insights, has some limitations that should be considered when interpreting its findings. Initially, the use of a small sample may restrict the generalization of conclusions. Additionally, analysis focused exclusively on a specific type of N95/PFF2 mask, of a particular brand and model, may restrict the applicability of results to that particular type. Finally, the lack of direct control by researchers over the storage of N95/PFF2 masks, despite the protocols and infrastructure provided by the institutions, may have introduced an unmonitored variable, with a potential impact on the results.

Despite these limitations, the use of visual inspection techniques and scanning electron microscopy made it possible to identify a significant gap in relation to the impact of reuse protocols for N95/PFF2 masks, especially regarding the integrity and functionality of the characteristics inherent to each layer of the devices after being subjected to reuse protocols for periods of seven and fifteen days. Therefore, the reuse of this equipment, despite being a recommendation from international and national health bodies, poses a tangible risk to the safety of health professionals.

The implications of this study for the advancement of scientific knowledge in the area of health and nursing are notable. By identifying alterations in the structural and morphological integrity of N95/PFF2 masks after reuse, the importance of reevaluating existing protocols and developing more effective guidelines is highlighted. Therefore, this research encourages additional testing to ensure adequate protection for health professionals.

## Conclusion

Seven- and fifteen-day reuse protocols potentially influenced the occurrence of morphological alterations in the fibers of the N95/PFF2 mask layers, in addition to the degradation of both the structural integrity and the fixation components. These findings suggest that the reuse of these respiratory devices in clinical practice leads to a significant reduction in the effectiveness of the equipment in terms of integrity and functionality of the sealing and filtration characteristics, therefore compromising the safety of health professionals.
