# Supplementary material for: Reuse of N95/PFF2 masks in clinical practice: morphological and structural analysis
Source: Rev Lat Am Enfermagem. 2024 Jul 5;32:e4209. doi: 10.1590/1518-8345.7045.4209 (PMC11251686; doi:10.1590/1518-8345.7045.4209)
Supplement: Translation

# Translation: Reutilización de mascarillas N95/PFF2 en la práctica clínica: análisis morfológico y estructural ^*^

**DOI:** 10.1590/1518-8345.7045.4208

**Authors:** Viviane Lopes Vimieiro, Claysson Bruno Santos Vimieiro, Adriana Cristina de Oliveira

**Affiliations:** ^1^ Universidade Federal de Minas Gerais, Escola de Enfermagem, Belo Horizonte, MG, Brasil.; ^2^ Becaria de la Fundação de Amparo à Pesquisa do Estado de Minas Gerais (FAPEMIG), Brasil.; ^3^ Pontifícia Universidade Católica de Minas Gerais, Belo Horizonte, MG, Brasil.; ^4^ Universidade Federal de Minas Gerais, Belo Horizonte, MG, Brasil.

**Keywords:** Equipo de Protección Personal, Respiradores N95, Equipo Reutilizado, Personal de Salud, Salud Laboral, Microscopía Electrónica de Rastreo

## Abstract

**Objetivo:**

analizar la integridad de mascarillas N95/PFF2 con relación a la morfología de las fibras, porosidad, grietas y microagujeros, así como identificar daños visibles en su estructura y componentes, luego de protocolos de reutilización de siete y quince días.

**Método:**

estudio transversal. Se analizaron las características estructurales y morfológicas de una mascarilla N95/PFF2 nueva en comparación con las mascarillas N95/PFF2 (n=10) utilizadas en protocolos de siete y quince días, mediante inspección visual y microscopía electrónica de barrido.

**Resultados:**

tras la inspección visual, siguiendo el protocolo de siete días, el 40% y el 60% de las mascarillas N95/PFF2 mostraron, respectivamente, marcas de identificación personal y suciedad externa e interna. Además, el 20% presentó aflojamiento y/o desgarro de las correas de sujeción, mientras que el 100% presentó algún tipo de daño en los clips nasales. En el protocolo de quince días, todas las mascarillas N95/PFF2 tenían suciedad, correas de sujeción sueltas y clips nasales dañados, y el 80% tenía pliegues. La microscopía electrónica reveló un aumento de poros y aflojamiento en las tramas a partir de los siete días, extendiéndose hasta los quince días, con presencia de microagujeros y residuos.

**Conclusión:**

la reutilización de las mascarillas N95/PFF2 compromete su integridad estructural y morfológica. Es crucial realizar pruebas para medir el impacto de esta práctica en la seguridad de los profesionales de la salud.

## Destacados

**(1)** Alteraciones morfológicas y estructurales en las mascarillas N95/PFF2 tras su reutilización.

**(2)** Impacto en la integridad y funcionalidad de las mascarillas N95/PFF2 tras su reutilización.

**(3)** Implicaciones para la seguridad de los profesionales de la salud.

**(4)** Necesidad de reevaluar los protocolos de reutilización.

**(5)** La Microscopía Electrónica de Barrido como herramienta valiosa.

## Introducción

 La mascarilla N95/PFF2 es el principal Equipo de Protección Personal (EPP) utilizado en la prevención y control de enfermedades respiratorias como tuberculosis, sarampión, COVID-19 e influenza H5N1 ^(^ ^1^ ^)^ . La integridad estructural de las mascarillas N95/PFF2 es esencial para garantizar el ajuste facial y una eficiencia de filtración mínima del 95% de las partículas en el aire de hasta 0,3 micras (μm) para garantizar la seguridad de los profesionales de la salud ^(^ ^2^ ^)^ . 

 La *Occupational Safety and Health Administration* (OSHA) exige que se utilice la mascarilla N95/PFF2 solo una vez y se deseche inmediatamente después de su uso ^(^ ^3^ ^)^ . Sin embargo, la escasez de equipos durante epidemias, como la de influenza H1N1 en 2009 y del Síndrome Respiratorio de Oriente Medio en 2012, y la pandemia de COVID-19, han llevado a organismos como el *Centers for Disease Control and Prevention* (CDC), la *Agência Nacional de Vigilância Sanitária* (ANVISA) y otras organizaciones de salud a recomendar la implementación de protocolos de reutilización limitada ^(^ ^4^ ^-^ ^6^ ^)^ . Estos protocolos incluyen el uso de mascarillas N95/PFF2 en múltiples atenciones o turnos de trabajo, y su retirada y almacenamiento para uso posterior ^(^ ^5^ ^)^ . 

 A pesar de ser justificables debido a la escasez del dispositivo, tales recomendaciones pueden comprometer la integridad estructural de las mascarillas N95/PFF2, resultando en posibles daños a la protección respiratoria ofrecida a los usuarios. De hecho, estudios realizados entre profesionales que trabajan en servicios de emergencia revelaron que las mascarillas N95/PFF2 reutilizadas durante más de tres turnos de ocho horas presentaban tasas de falla considerablemente altas (≥ 40%) en las pruebas de sellado, fenómeno posiblemente atribuido a un ajuste inadecuado del EPP al rostro ^(^ ^7^ ^-^ ^8^ ^)^ . 

 Los riesgos adicionales asociados con los protocolos de reutilización incluyen la necesidad, inherente a la práctica clínica, de que los profesionales de la salud tomen descansos para ir al baño, comer e incluso descansar la cara. Estas circunstancias implican un mayor número de paramentaciones y desparamentaciones, lo que puede provocar el deterioro de los componentes de las mascarillas N95/PFF2, como aflojamiento o rotura de las correas de sujeción, rotura del clip nasal, pliegues, grietas y desgarros ^(^ ^9^ ^-^ ^10^ ^)^ . 

 Las mascarillas N95/PFF2 suelen estar compuestas de cuatro capas distintas: dos capas intermedias responsables de la filtración y del soporte, y capas exteriores e interiores que no filtran partículas ^(^ ^11^ ^-^ ^12^ ^)^ . La eficacia de filtración de una mascarilla N95/PFF2 está directamente relacionada con la preservación de las características morfológicas de las fibras de cada capa. Sin embargo, es posible que los profesionales de la salud no detecten la degradación del material del filtro, lo que puede provocar una reducción potencial de la protección respiratoria ^(^ ^11^ ^)^ . 

 Estudios anteriores sobre los impactos de la reutilización de mascarillas N95/PFF2 se basaron principalmente en pruebas de sellado y realización de inspecciones visuales ^(^ ^8^ ^-^ ^10^ ^,^ ^13^ ^-^ ^15^ ^)^ . No obstante, estos métodos pueden resultar insuficientes cuando se busca una evaluación objetiva de la eficacia de las mascarillas N95/PFF2. Además, la falta de directrices claras sobre el tiempo máximo de reutilización deja una laguna en el conocimiento. 

 Para abordar esta laguna, este estudio adopta una estrategia integral que combina la inspección visual tradicional con técnicas avanzadas como la microscopía electrónica de barrido (MEB). Este enfoque permite un análisis más detallado y preciso, capaz de identificar daños morfológicos microscópicos que pueden no ser perceptibles a simple vista ^(^ ^16^ ^-^ ^17^ ^)^ . 

Debido a la escasez de evidencia sobre el tiempo seguro de reutilización de las mascarillas N95/PFF2 que garantice la preservación de la integridad estructural, este estudio tiene como objetivo analizar la integridad de las mascarillas N95/PFF2 con relación a la morfología de las fibras, la porosidad, las grietas y los microagujeros, así como identificar daños visibles en su estructura y componentes luego de protocolos de reutilización de siete y de quince días.

## Método

### Diseño del estudio y escenario

 Estudio transversal. El escenario incluyó dos Unidades de Cuidados Intensivos (UCI) ubicadas en dos hospitales públicos de Belo Horizonte, MG, Brasil, con capacidad de 10 y 54 camas, respectivamente. La elección de estas instituciones se basó en la diferencia de los protocolos de reutilización de las mascarillas N95/PFF2 adoptados, siendo de siete días para la primera y quince días para la segunda. Cada unidad contaba con espacios exclusivos, aireados y ventilados destinados al almacenamiento seguro de estos dispositivos. Los protocolos de reutilización fueron elaborados por el *Setor de Segurança do Trabalho* (SST) y por la *Comissão de Controle de Infecção Hospitalar* (CCIH) de cada hospital, con orientaciones detalladas para los profesionales de la salud sobre el embalaje correcto, destacando la importancia del uso de papel adecuado y una identificación clara de cada mascarilla N95/PFF2 con nombre de usuario y tiempo de uso. 

### Población

La población elegible estuvo compuesta por profesionales de enfermería que trabajan en cuidados directos en las unidades seleccionadas.

### Criterios de selección

Se incluyeron mascarillas N95/PFF2 utilizadas por los profesionales de enfermería durante el turno de día, de acuerdo con los protocolos de reutilización (siete y quince días). La elección de este turno se debió al mayor número de procedimientos realizados durante este período. Se excluyeron las mascarillas N95/PFF2 utilizadas por profesionales que no siguieron los intervalos de tiempo determinados por los protocolos de reutilización.

### Definición de la muestra

 La muestra de conveniencia se dividió en dos grupos de comparación: un grupo con cinco mascarillas N95/PFF2 utilizadas durante siete días, y el otro con cinco utilizadas durante quince días, ambos siguiendo el protocolo de reutilización establecido. Es importante resaltar que todas las mascarillas N95/PFF2 eran de la misma marca y modelo, con un tamaño estándar. Este modelo en particular se caracteriza por sus dos correas de sujeción de material elástico, clip nasal de metal sin revestimiento para un ajuste personalizado y diseño plegable. Además, todos los dispositivos contaban con un Certificado de Aprobación (CA) emitido por el *Ministério do Trabalho e Emprego* (MTE) de Brasil, garantizando que cumplían con los rigurosos estándares de seguridad y calidad exigidos por la legislación brasileña. 

### Variables del estudio

 Las variables del estudio fueron analizadas mediante dos instrumentos de recolección de datos, ambos desarrollados por los investigadores y basados en las normas de la *Occupational Safety and Health Administration* de EE. UU y del *Ministério do Trabalho e Emprego* de Brasil sobre el uso seguro de la mascarilla N95/PFF2. El primer instrumento evaluó la integridad estructural de las mascarillas N95/PFF2, considerando el tiempo de uso (siete o quince días), la categoría profesional (enfermeros o técnicos de enfermería), y la presencia de marcas de identificación personal, pliegues, agujeros o desgarros en la superficie, manchas y suciedad en las partes externa e interna (con respuestas de sí o no para cada ítem). En los casos afirmativos, se especificaron los tipos de manchas o suciedad, incluyendo restos de maquillaje, manchas amarillas u oscuras, café, garabatos con bolígrafo, entre otros. Para cuantificar la extensión de la suciedad o de las manchas, se adoptó una estimación en múltiplos del 25% del área comprometida de la mascarilla N95/PFF2. Este enfoque incluyó una evaluación visual, seguida de la clasificación de las áreas afectadas en categorías, según su extensión. Además, se evaluó el estado de conservación de las correas de sujeción (intactas o estiradas) y del clip nasal (intacto, aplastado o con marca de dobleces, roto, con ranuras u otras condiciones especificadas). 

El segundo instrumento se centró en las características morfológicas de las fibras de las diferentes capas de las mascarillas N95/PFF2, incluyendo el tiempo de uso (siete o quince días), la categoría profesional (enfermeros o técnicos de enfermería), las capas específicas (externa, estructural, filtrante e interna), las características de las fibras (bien conservadas, irregulares o muy irregulares), la presencia de imperfecciones (microagujeros, grietas o ambos) y residuos. La región elegida para la recolección de la muestra fue la zona cercana a la parte nasal, en el lado derecho de la mascarilla N95/PFF2, seleccionada por el mayor grado de humedad provocado por la respiración del usuario.

### Período, instrumentos utilizados para recolectar la información y recolección de datos

La recolección de datos se realizó entre los meses de febrero y abril de 2023. Diariamente, al final de cada turno diurno, se recogían mascarillas N95/PFF2 según un cronograma previamente establecido en conjunto con los servicios de salud. Estas muestras estaban acondicionadas en sobres marrones, etiquetados con información sobre el lugar y fecha de recolección, marca del dispositivo, período de uso y categoría profesional del usuario. Posteriormente, eran almacenadas en cajas rígidas que habían sido higienizadas con agua y jabón, en un ambiente ventilado. Durante el proceso se adoptaron estrictas medidas de seguridad, incluido el uso de delantales, guantes de procedimiento y mascarillas quirúrgicas.

 La investigadora era responsable del transporte de las mascarillas N95/PFF2, ya acondicionadas en las cajas, desde las unidades de cuidados intensivos hasta el Centro de Ingeniería de la *Pontifícia Universidade Católica de Minas Gerais* (PUC Minas), en Belo Horizonte, Brasil. En el Centro, la integridad estructural se analizó mediante inspección visual con luz natural. Se utilizaba una lente óptica con diez aumentos (Tomshin S.A., São Paulo, SP, Brasil) sobre una mesada desinfectada con alcohol al 70% para detectar la presencia de marcas de identificación, pliegues, agujeros o desgarros, manchas y suciedad, además de evaluar el estado de conservación de los componentes de la mascarilla N95/PFF2. Las marcas de identificación indicaban marcaciones realizadas por el usuario, generalmente con un bolígrafo, para distinguir su dispositivo y controlar el tiempo de uso. Los resultados se registraron en formularios individuales para cada muestra. 

 Después de la recolección e inspección visual, todas las mascarillas N95/PFF2 fueron enviadas al Laboratorio de Microscopía Avanzada de la *PUC Minas* . En este laboratorio, se utilizó un microscopio electrónico de barrido (JSM-6610LV, Jeol, Tokio, Japón) para analizar la morfología de las fibras en las diferentes capas del dispositivo. La manipulación se realizó con guantes y delantal no esterilizados sobre una mesada. Para el análisis, las muestras se seccionaron cerca de la región de la nariz, en el lado derecho, utilizando una tijera previamente desinfectada con alcohol al 70%. Las secciones tenían unas dimensiones aproximadas de 1,0 x 1,0 cm. Luego, las capas se separaron y se fijaron directamente a un “ *stub* ” de aluminio con cinta adhesiva de carbono de doble faz. Para que las superficies fueran conductoras para la visualización en el MEB, se depositó una película de oro de quince nanómetros de espesor sobre las muestras, utilizando un sistema de recubrimiento al vacío de alta presión (Denton Vacuum Desk S.A, Nueva Jersey, Estados Unidos de América). Las micrografías se obtuvieron con aumentos de 100x, 500x y 1000x. Cabe señalar que, poco después de llegar al Centro de Ingeniería y al Laboratorio de Microscopía, las mascarillas N95/PFF2 fueron inmediatamente sometidas a las pruebas. Este procedimiento se adoptó para evitar la influencia de cualquier variable ambiental derivada del almacenamiento en lugares inadecuados sobre los resultados. 

### Procesamiento y análisis de los datos

 Uso del *Statistical Package for the Social Sciences* (SPSS), versión 28.0, para análisis descriptivos y presentación de datos en tablas de distribución de frecuencias absolutas y relativas. 

### Aspectos éticos

El estudio siguió los principios éticos de la Resolución 466/2012 del Consejo Nacional de Salud, referente a investigaciones con seres humanos, recibiendo la aprobación del Comité de Ética en Investigación (CEP, por sus siglas en portugués) de Brasil, bajo dictamen número: 5.824.571 - Certificado de Presentación de Evaluación Ética (CAAE, por sus siglas en portugués): 65232922.8.0000.5149. Se invitó a los profesionales de enfermería a participar de forma voluntaria y se proporcionó toda la información pertinente, asegurando la firma individual del Término de Consentimiento Libre e Informado (TCLE, por sus siglas en portugués). Para preservar el anonimato, el proceso de recolección de datos fue diseñado para excluir cualquier dato que pudiera identificar a los individuos participantes.

## Resultados

 La integridad de las mascarillas N95/PFF2 con relación al protocolo de reutilización (siete y quince días) se evaluó mediante inspección visual con ayuda de una lente óptica de diez aumentos marca Tomshin ^®^ ( Figura 1 ). 

**Figura 1:** - Alteraciones estructurales en mascarillas N95/PFF2 después de su uso en protocolos de siete (n=5) y quince días (n=5), en un turno de 12 horas. Belo Horizonte, MG, Brasil, 2023

En cuanto a la estructura de las mascarillas N95/PFF2, se observó la presencia de marcas de identificación en el 40% (2/5) de los dispositivos que fueron reutilizados durante siete días. Estas marcas, realizadas por técnicos de enfermería, consistieron en escribir las iniciales de sus nombres y la fecha de recibimiento, utilizando bolígrafos, en el exterior de las mascarillas N95/PFF2. El objetivo de estas marcas era identificar y controlar el tiempo de uso de cada equipo.

Respecto a la suciedad, tras siete días de uso el 40% (2/5) de las mascarillas N95/PFF2 presentaban manchas amarillas u oscuras de origen indeterminado en al menos el 25% de su superficie en la capa exterior, mientras que el 60% (3/5) tenía suciedad en la capa interior. En cuanto a las muestras utilizadas durante quince días, todas ellas presentaban suciedad en ambas capas, ocupando las manchas una superficie inferior al 25% en la capa exterior, y en la interior la mayoría, el 60% (3/5), presentaba restos de maquillaje en una superficie superior al 75%.

Respecto a los componentes de las mascarillas N95/PFF2 reutilizadas durante siete días, el 20% (1/5) de las correas de sujeción presentaron aflojamiento y el 20% (1/5) se rompieron; el 100% (n=5) de los clips nasales presentaban algún tipo de daño: el 20% (1/5) estaban abollados o presentaban marcas de pliegues, el 20% (1/5), además de este daño, también presentaban ranuras, y el 60% (3/5) presentaban únicamente ranuras. En todas las mascarillas N95/PFF2 reutilizadas durante quince días las correas de sujeción estaban aflojadas y el clip nasal estaba comprometido, con abolladuras o marcas de pliegues y ranuras. Además, en el 40% (2/5) de las muestras del protocolo de quince días se observaron defoliación entre las capas del interior y presencia de apósitos de espuma insertados por profesionales de la salud para protección nasal.

Todas las mascarillas N95/PFF2 fueron sometidas a microscopía electrónica de barrido para analizar las alteraciones morfológicas en las fibras de cada capa, luego de ser utilizadas en protocolos de reutilización. Este análisis reveló que todas las muestras presentaron modificaciones morfológicas similares. Dada esta coherencia en los hallazgos, se seleccionó una mascarilla N95/PFF2 representativa de cada protocolo para ilustrarla en las figuras del estudio. En comparación, la mascarilla N95/PFF2 de control (sin usar) mostró en las micrografías una superficie de contorno de fibra bien conservada, sin microagujeros, grietas ni residuos adheridos.

 Al examinar las capas externas de las muestras de siete días, se observó la presencia de poros entre los tejidos y residuos adheridos a las fibras. En las muestras de quince días se observaron microagujeros, hebras de fibras sueltas, así como residuos adheridos a las fibras y poros entre los tejidos ( Figura 2 ). 

**Figura 2:** - Microscopía electrónica de barrido de la capa exterior de la mascarilla N95/PFF2 de control y de las muestras en los protocolos de reutilización (siete y quince días) durante turnos de 12 horas, con aumentos de 100x, 500x y 1000x. Belo Horizonte, MG, Brasil, 2023

Las imágenes microscópicas 2(A-C) de la capa exterior de la mascarilla N95/PFF2 de control revelaron fibras bien conservadas. En las imágenes 2(D-F), de las muestras de siete días, se puede observar la presencia de poros entre las tramas en 2(D) y 2(F) y residuos en 2(E). En las imágenes 2(G-I), correspondientes a las muestras de quince días, se identificaron microagujeros y fibras sueltas en 2(G), residuos en 2(H) y 2(I), y poros entre las tramas en 2(I).

 En el análisis de las capas estructurales de las mascarillas N95/PFF2 después de los protocolos de reutilización de siete días, se observaron tramas “enredadas”, roturas de fibras y residuos adheridos a las mismas. En las muestras reutilizadas durante quince días, además de las tramas “enredadas” y residuos adheridos, también se identificaron desgastes en las fibras ( Figura 3 ). 

 Las imágenes microscópicas 3(A-C) de la mascarilla N95/PFF2 de control mostraron fibras bien conservadas. La imagen 3(D) de las muestras de siete días reveló tramas “enredadas”, la imagen 3(E) mostró roturas de fibras y residuos, mientras que la imagen 3(F) mostró solo residuos. Las imágenes 3(G-I) de las muestras de quince días revelaron tramas “enredadas” en 3(G), residuos en 3(H) y residuos con desgaste de fibras en 3(I). Respecto a las capas filtrantes de las mascarillas N95/PFF2 sometidas a los protocolos de reutilización de siete y quince días, se observó la presencia de tramas “enredadas” y roturas de fibras. En las muestras de quince días, además de las tramas “enredadas”, se encontraron poros más grandes entre las redes ( Figura 4 ). 

**Figura 3:** - Microscopía electrónica de barrido de la capa estructural de la mascarilla N95/PFF2 de control y de las muestras en los protocolos de reutilización (siete y quince días) durante turnos de 12 horas, con aumentos de 100x, 500x y 1000x. Belo Horizonte, MG, Brasil, 2023

**Figura 4:** - Microscopía electrónica de barrido de la capa filtrante de la mascarilla N95/PFF2 de control y de las muestras en los protocolos de reutilización (siete y quince días) durante turnos de 12 horas, con aumentos de 100x, 500x y 1000x. Belo Horizonte, MG, Brasil, 2023

Las imágenes 4(A-C) de la mascarilla N95/PFF2 de control mostraron fibras bien conservadas. Las imágenes 4(D-F) de las muestras de siete días revelaron tramas “enredadas”. Las imágenes 4(G-H) de las muestras de quince días también revelaron tramas “enredadas”, mientras que la imagen 4(I) reveló poros entre las tramas.

 Al analizar las capas internas de las mascarillas N95/PFF2 utilizadas en los protocolos de siete días, se identificaron microagujeros, residuos adheridos a las fibras y poros entre las tramas. En las muestras reutilizadas en los protocolos de quince días, además de estos daños, se reveló la presencia de hebras de fibras sueltas ( Figura 5 ). 

**Figura 5:** - Microscopía electrónica de barrido de la capa interna de la mascarilla N95/PFF2 de control y de las muestras en los protocolos de reutilización (siete y quince días) durante turnos de 12 horas, con aumentos de 100x, 500x y 1000x. Belo Horizonte, MG, Brasil, 2023

Las imágenes 5(A-C) de la mascarilla N95/PFF2 de control mostraron fibras bien conservadas. La imagen 5(D) de las muestras de siete días reveló la presencia de microagujeros, residuos y poros entre las tramas, mientras que las imágenes 5(E) y 5(F) revelaron poros entre las tramas. La imagen 5(G) de las muestras de quince días también reveló poros entre las tramas, y las imágenes 5(H) y 5(I) mostraron residuos y poros entre las tramas, así como hebras de fibras sueltas.

## Discusión

En el análisis de mascarillas N95/PFF2 sometidas a protocolos de reutilización por períodos de siete y quince días, se encontraron alteraciones sustanciales atribuibles a esta práctica, afectando tanto la integridad estructural como la morfología de las fibras. Este hecho sugiere un mayor riesgo para la seguridad de los profesionales de la salud, destacando la necesidad de un enfoque cauteloso y sensato a la hora de reutilizar estos equipos de protección.

 Estos hallazgos son de gran relevancia, dado que la implementación de estos protocolos de reutilización fue fundamental para satisfacer la alta demanda global durante la pandemia de COVID-19, así como en epidemias anteriores, como la de influenza H1N1 en 2009 y del Síndrome Respiratorio de Oriente Medio en 2012 ^(^ ^18^ ^-^ ^21^ ^)^ . Asimismo, esta estrategia todavía se adopta como política para reducir los costos financieros en varias instituciones hospitalarias alrededor del mundo, especialmente en países que enfrentan impactos desproporcionados de la COVID-19, como Brasil, India y China, con altos números de casos y mortalidad, además de una alta incidencia de otras enfermedades respiratorias, como la tuberculosis ^(^ ^22^ ^-^ ^24^ ^)^ . 

 Al evaluar la integridad de las mascarillas N95/PFF2 reutilizadas durante siete y quince días, se identificaron, mediante inspección visual, varios signos de desgaste en las correas de sujeción y degradación en los clips nasales. Estos daños corroboran los hallazgos de estudios realizados tanto en ambiente clínico como de laboratorio, destacando la fragilidad de estos equipos en condiciones de reutilización ^(^ ^9^ ^,^ ^25^ ^)^ . Una investigación clínica, realizada en un hospital terciario de la India durante la pandemia de COVID-19, indicó que el 9% de los profesionales de la salud informaron que el aflojamiento de las correas de sujeción, generalmente después de cinco reutilizaciones en turnos de ocho horas, era una de las razones principales para la eliminación temprana de mascarillas N95/PFF2 ^(^ ^25^ ^)^ . En un contexto de laboratorio, simulando un turno de diez horas, se encontró que la ruptura de cinco clips nasales y de ocho elementos de fijación, que ocurrió durante 20 episodios de paramentación y desparamentación, resultó en el fracaso de las pruebas de sellado para todos los participantes que usaban tales dispositivos ^(^ ^9^ ^)^ . 

 La degradación de los componentes de las mascarillas N95/PFF2 puede verse exacerbada por la intensa rutina de los profesionales de la salud, que muchas veces necesitan paramentarse y desparamentarse varias veces durante la jornada laboral, especialmente para pausas esenciales como la hidratación, la alimentación e incluso el descanso facial ^(^ ^9^ ^,^ ^15^ ^)^ . Investigaciones realizadas en los servicios de salud muestran una variación en la frecuencia de paramentaciones, oscilando entre hasta cinco ocurrencias en turnos de diez horas y un promedio de cuarenta en turnos de doce horas ^(^ ^26^ ^-^ ^27^ ^)^ . Por lo tanto, es vital que los usuarios realicen inspecciones visuales rigurosas y verifiquen el sellado de las mascarillas N95/PFF2 antes de cada uso para garantizar que se mantenga la integridad de estos dispositivos y, en consecuencia, garantizar una protección eficaz contra agentes infecciosos transmitidos por el aire ^(^ ^4^ ^-^ ^5^ ^,^ ^28^ ^)^ . Sin embargo, un estudio reciente reveló que el 57% de los enfermeros no realizan estos controles, destacando la urgente necesidad de una formación frecuente y una mayor concientización sobre la esencialidad de estos procedimientos para el uso correcto y seguro de los equipos de protección personal ^(^ ^4^ ^,^ ^29^ ^)^ . 

 Otro punto crítico identificado en el presente estudio fue la alarmante incidencia de suciedad en las mascarillas N95/PFF2, realidad que va en contra de las directrices sanitarias, que recomiendan la sustitución inmediata de dispositivos sucios, húmedos o dañados ^(^ ^2^ ^,^ ^4^ ^)^ . Esta observación es corroborada por una investigación realizada en un ambiente hospitalario, en la que la inspección visual reveló la presencia de manchas o suciedad en todas las mascarillas N95/PFF2 utilizadas por los auxiliares de enfermería después de cinco reutilizaciones, siendo los residuos cosméticos los más comunes ^(^ ^10^ ^)^ . Estos hallazgos resaltan la necesidad crítica de cumplir con las prácticas recomendadas por las agencias de salud, en términos de inspección y mantenimiento de equipos libres de contaminación y suciedad, así como intactos para garantizar la protección efectiva de los profesionales de la salud. Sin embargo, cabe señalar que el uso de maquillaje intensifica el deterioro de las fibras, lo que, a su vez, conduce a una disminución de su eficacia ^(^ ^4^ ^-^ ^5^ ^)^ . 

 Las instituciones hospitalarias participantes en el estudio, de acuerdo con las directrices establecidas por las autoridades de salud ANVISA y CDC, proporcionaron lugares propios, limpios y ventilados para almacenar las mascarillas N95/PFF2 ^(^ ^2^ ^,^ ^4^ ^)^ . Además, promovieron el uso de sobres de papel, que facilitan la circulación del aire y permiten la identificación personal, evitando marcas directas en los dispositivos. Estas prácticas tienen como objetivo preservar la integridad estructural del EPP y reducir el riesgo de cambios involuntarios entre usuarios. Sin embargo, después de los protocolos de siete y quince días, se observaron en las muestras pliegues y marcas realizadas por un profesional con bolígrafos, lo que sugiere un posible acondicionamiento inadecuado de las mascarillas N95/PFF2 en bolsillos de la ropa o en objetos personales, como bolsos. Esta condición también se observa en otros estudios, lo que llama la atención sobre la importancia de implementar acciones de sensibilización para desalentar el almacenamiento inadecuado de estos dispositivos ^(^ ^25^ ^,^ ^27^ ^)^ . 

 Otro aspecto que merece ser destacado en el contexto del protocolo de reutilización de mascarillas N95/PFF2 es la aparición de eventos adversos, entre ellos lesiones por presión en el puente nasal por fricción y cizallamiento provocados por el desplazamiento del dispositivo ^(^ ^30^ ^-^ ^31^ ^)^ . En la práctica clínica, es común que los profesionales de la salud utilicen adhesivos de silicona o espuma en el interior de las mascarillas N95/PFF2 para proteger la piel contra daños y brindar comodidad en la región, especialmente en la nariz, debido a la estructura metálica del clip nasal ^(^ ^32^ ^-^ ^33^ ^)^ . Estas estrategias se observaron en dos muestras reutilizadas en protocolos de quince días en este estudio. Sin embargo, estos apósitos de espuma pueden comprometer el ajuste de estos dispositivos, debido a la posibilidad de que queden espacios entre la piel y el equipo de protección respiratoria ^(^ ^32^ ^)^ . 

 Las mascarillas N95/PFF2, certificadas por el *National Institute for Occupational Safety and Health* (NIOSH), están fabricadas con microfibras sintéticas de polipropileno, distribuidas en cuatro capas distintas: externa, estructural, filtrante e interna ^(^ ^28^ ^,^ ^34^ ^)^ . Las capas exterior e interior están diseñadas específicamente para proteger contra la humedad, con diámetros de fibra entre 20 y 25 micras ^(^ ^34^ ^)^ . Por otro lado, el medio filtrante está compuesto por fibras más pequeñas, de dos a cinco micras, tratadas electrostáticamente para retener al menos el 95% de las partículas en el aire de hasta 0,3 µm ^(^ ^34^ ^)^ . 

A partir del séptimo día de uso de las mascarillas N95/PFF2, los análisis de microscopía electrónica de barrido revelaron una tendencia a aumentar los poros en la mayoría de las capas y la presencia de tramas “enredadas” en las capas estructural y filtrante, posiblemente resultantes del desprendimiento de las fibras, creando espacios más grandes entre ellos. Estas alteraciones se intensificaron en el protocolo de uso de quince días, destacando la relación directa entre el tiempo de reutilización y la magnitud de los resultados observados.

 En el contexto de la salud ocupacional, estos cambios morfológicos en las fibras de los dispositivos generan preocupación, ya que aumentan sustancialmente la posibilidad de que sangre, secreciones respiratorias o nasales y otros fluidos corporales penetren a través de las capas de las mascarillas N95/PFF2. Este escenario es especialmente crítico en entornos como las unidades de cuidados intensivos, donde los procedimientos que generan aerosoles son rutinarios y presentan un alto riesgo de transmisión de virus respiratorios ^(^ ^35^ ^-^ ^36^ ^)^ . 

 Además, un estudio adicional destaca que las mascarillas N95/PFF2 con poros más pequeños, más cercanas a sus características originales, ofrecen una eficiencia de filtración superior ^(^ ^37^ ^)^ . Esto significa que mayores espacios entre las tramas favorecen la penetración de un mayor número de partículas aerosolizadas, aumentando la vulnerabilidad de estos profesionales ^(^ ^37^ ^-^ ^38^ ^)^ . 

Las micrografías de las mascarillas N95/PFF2 también mostraron la presencia de microagujeros y la rotura de las fibras, posiblemente como resultado del deshilachado, lo que conduce a la expansión de los espacios de retención de partículas y facilita la acumulación de residuos entre las fibras. Los elementos observados durante la inspección visual, como suciedad, pliegues y marcas de identificación personal en las muestras, pueden haber influido en estas alteraciones morfológicas. Por tanto, es crucial establecer protocolos adecuados y límites de reutilización de estos dispositivos para garantizar la protección necesaria a los profesionales de la salud.

Este estudio, a pesar de sus valiosos conocimientos, tiene algunas limitaciones que deben tenerse en cuenta al interpretar sus hallazgos. Inicialmente, el uso de una muestra pequeña puede restringir la generalización de las conclusiones. Asimismo, el análisis centrado exclusivamente en un tipo específico de mascarilla N95/PFF2, de una marca y modelo específicos, puede restringir la aplicabilidad de los resultados a ese tipo en particular. Finalmente, la falta de control directo por parte de los investigadores sobre el almacenamiento de mascarillas N95/PFF2, a pesar de los protocolos e infraestructura proporcionados por las instituciones, puede haber introducido una variable no monitoreada, con potencial impacto en los resultados.

Pese a estas limitaciones, el uso de técnicas de inspección visual y la microscopía electrónica de barrido permitió identificar una laguna significativa con relación al impacto de los protocolos de reutilización de las mascarillas N95/PFF2, especialmente en lo que respecta a la integridad y funcionalidad de las características inherentes a cada capa de los dispositivos tras ser sometidos a protocolos de reutilización durante períodos de siete y quince días. Por ende, la reutilización de estos equipos, aunque sea una recomendación de organismos sanitarios nacionales e internacionales, supone un riesgo tangible para la seguridad de los profesionales de la salud.

Son notables las implicaciones de este estudio para el avance del conocimiento científico en el área de la salud y la enfermería. Al identificar alteraciones en la integridad estructural y morfológica de las mascarillas N95/PFF2 después de su reutilización, se destaca la importancia de reevaluar los protocolos existentes y desarrollar pautas más efectivas. Por ello, esta investigación fomenta pruebas adicionales para garantizar una protección adecuada para los profesionales de la salud.

## Conclusión

Los protocolos de reutilización de siete y quince días influyeron potencialmente en la aparición de alteraciones morfológicas en las fibras de las capas de las mascarillas N95/PFF2, además de la degradación tanto de la integridad estructural como de los componentes de fijación. Estos hallazgos sugieren que la reutilización de estos dispositivos respiratorios en la práctica clínica conduce a una reducción significativa de la eficacia del equipo en términos de integridad y funcionalidad de las características de sellado y filtración, comprometiendo así la seguridad de los profesionales de la salud.

# Translation: Reutilização das máscaras N95/PFF2 na prática clínica: análise morfológica e estrutural ^*^

**DOI:** 10.1590/1518-8345.7045.4210

**Authors:** Viviane Lopes Vimieiro, Claysson Bruno Santos Vimieiro, Adriana Cristina de Oliveira

**Affiliations:** ^1^ Universidade Federal de Minas Gerais, Escola de Enfermagem, Belo Horizonte, MG, Brasil.; ^2^ Bolsista da Fundação de Amparo à Pesquisa do Estado de Minas Gerais (FAPEMIG), Brasil.; ^3^ Pontifícia Universidade Católica de Minas Gerais, Belo Horizonte, MG, Brasil.; ^4^ Universidade Federal de Minas Gerais, Belo Horizonte, MG, Brasil.

**Keywords:** Equipamento de Proteção Individual, Respiradores N95, Reutilização de Equipamento, Pessoal de Saúde, Saúde Ocupacional, Microscopia Eletrônica de Varredura

## Abstract

**Objetivo:**

analisar a integridade das máscaras N95/PFF2 em relação à morfologia das fibras, porosidade, fissuras e micro furos, bem como identificar danos visíveis em sua estrutura e componentes, após protocolos de reutilização de sete e quinze dias.

**Método:**

estudo transversal. Características estruturais e morfológicas de uma máscara N95/PFF2 nova foram analisadas em comparação com máscaras N95/PFF2 (n=10) utilizadas em protocolos de sete e quinze dias, por meio da inspeção visual e microscopia eletrônica de varredura.

**Resultados:**

na inspeção visual, seguindo o protocolo de sete dias, 40% e 60% das máscaras N95/PFF2 apresentaram, respectivamente, marcas de identificação pessoal e sujidades externas e internas. Além disso, 20% exibiram afrouxamento e/ou rompimento das tiras de fixação, enquanto 100% mostraram algum tipo de dano nos clipes nasais. No protocolo de quinze dias, todas as máscaras N95/PFF2 apresentavam sujidade, tiras de fixação frouxas e clipes nasais comprometidos; 80% possuíam dobras. A microscopia eletrônica revelou aumento dos poros e afrouxamento nas tramas a partir de sete dias, ampliando-se até quinze dias, com presença de micro furos e detritos.

**Conclusão:**

a reutilização das máscaras N95/PFF2 compromete a integridade estrutural e morfológica. É crucial realizar testes para mensurar o impacto dessa prática na segurança dos profissionais de saúde.

## Destaques

**(1)** Alterações morfológicas e estruturas das máscaras N95/PFF2 após reutilização.

**(2)** Impacto na integridade e funcionalidade das máscaras N95/PFF2 após reutilização.

**(3)** Implicações para a segurança dos profissionais de saúde.

**(4)** Necessidade de reavaliação dos protocolos de reutilização.

**(5)** Microscopia Eletrônica de Varredura como uma ferramenta valiosa.

## Introdução

 A máscara N95/PFF2 é o principal Equipamento de Proteção Individual (EPI) utilizado na prevenção e controle de doenças respiratórias como tuberculose, sarampo, COVID-19 e influenza H5N1 ^(^ ^1^ ^)^ . A integridade estrutural das máscaras N95/PFF2 é essencial para garantir a adaptação facial e uma eficiência mínima de filtração de 95% de partículas transportadas pelo ar de até 0,3 mícrons (µm), a fim de assegurar a segurança dos profissionais de saúde ^(^ ^2^ ^)^ . 

 A *Occupational Safety and Health Administration* (OSHA) estabelece que a máscara N95/PFF2 seja utilizada apenas uma vez e descartada imediatamente após o uso ^(^ ^3^ ^)^ . Contudo, a escassez do equipamento durante epidemias, como a influenza H1N1 em 2009, Síndrome Respiratória do Oriente Médio em 2012 e a pandemia de COVID-19, levou órgãos como o *Centers for Disease Control and Prevention* (CDC), a Agência Nacional de Vigilância Sanitária (ANVISA), e outras organizações de saúde a recomendarem a implementação de protocolos de reutilização limitada ^(^ ^4^ ^-^ ^6^ ^)^ . Esses protocolos incluem a utilização das máscaras N95/PFF2 em múltiplos atendimentos ou turnos de trabalhos, seguidos por sua remoção e armazenamento para uso subsequente ^(^ ^5^ ^)^ . 

 Apesar de serem justificáveis devido à escassez do dispositivo, tais recomendações podem comprometer a integridade estrutural das máscaras N95/PFF2, resultando em possíveis prejuízos à proteção respiratória oferecida aos usuários. De fato, estudos conduzidos entre profissionais que atuam em serviços de emergência mostraram que máscaras N95/PFF2 reutilizadas por mais de três turnos de oito horas apresentaram taxas consideravelmente altas de falha (≥ 40%) nos testes de vedação, fenômeno possivelmente atribuído ao ajuste inadequado do EPI ao rosto ^(^ ^7^ ^-^ ^8^ ^)^ . 

 Riscos adicionais associados aos protocolos de reutilização englobam a necessidade inerente à prática clínica de que os profissionais de saúde façam pausas para tarefas como uso de banheiro, refeições e até descanso da face. Estas circunstâncias implicam em um maior número de paramentações e desparamentações, que podem conduzir à deterioração dos componentes das máscaras N95/PFF2, como o afrouxamento ou rompimento das tiras de fixação, quebras do clipe nasal, dobras, fissuras e rasgos ^(^ ^9^ ^-^ ^10^ ^)^ . 

 As máscaras N95/PFF2 são frequentemente constituídas por quatro camadas distintas: duas camadas intermediárias responsáveis pela filtragem e sustentação, e camadas externas e internas que não filtram partículas ^(^ ^11^ ^-^ ^12^ ^)^ . A eficácia de filtração de uma máscara N95/PFF2 está diretamente relacionada à preservação das características morfológicas das fibras em cada camada. No entanto, a degradação do material filtrante pode não ser detectada pelos profissionais de saúde, resultando em potencial redução na proteção respiratória ^(^ ^11^ ^)^ . 

 Estudos anteriores sobre o impacto da reutilização das máscaras N95/PFF2 basearam-se principalmente em testar a vedação e realizar a inspeção visual ^(^ ^8^ ^-^ ^10^ ^,^ ^13^ ^-^ ^15^ ^)^ . No entanto, esses métodos podem ser insuficientes quando se busca uma avaliação objetiva da eficácia das máscaras N95/PFF2. Além disso, a falta de diretrizes claras quanto ao tempo máximo de reuso deixa uma lacuna no conhecimento. 

 Para abordar essa lacuna, este estudo adota uma estratégia abrangente que combina a inspeção visual tradicional com técnica avançada, como a microscopia eletrônica de varredura (MEV). Essa abordagem permite uma análise mais detalhada e precisa, capaz de identificar danos morfológicos microscópicos que podem não ser visualizados a olho nu ^(^ ^16^ ^-^ ^17^ ^)^ . 

Devido à escassez de evidências sobre o tempo seguro de reutilização das máscaras N95/PFF2, que assegure a preservação da integridade estrutural, este estudo tem como objetivo analisar a integridade das máscaras N95/PFF2 em relação à morfologia das fibras, porosidade, fissuras e micro furos, bem como identificar danos visíveis em sua estrutura e componentes, após protocolos de reutilização de sete e quinze dias.

## Método

### Tipo e local de estudo

Estudo transversal. O local do estudo foram duas Unidades de Terapia Intensiva (UTI) localizadas em dois hospitais públicos em Belo Horizonte, MG, Brasil, com capacidade de 10 e 54 leitos, respectivamente. A escolha dessas instituições baseou-se na diferença dos protocolos de reutilização das máscaras N95/PFF2 adotados, sendo de sete dias para a primeira e quinze dias para a segunda. Cada unidade tinha locais exclusivos, arejados e ventilados, destinados ao armazenamento seguro desses dispositivos. Os protocolos de reutilização foram elaborados pelo Setor de Segurança do Trabalho (SST) e pela Comissão de Controle de Infecção Hospitalar (CCIH) de cada hospital, incluindo orientações detalhadas para os profissionais de saúde sobre o correto acondicionamento, ressaltando a importância da utilização de papel apropriado e da identificação clara de cada máscara N95/PFF2 com nome do usuário e tempo de uso.

### População

A população elegível consistiu nos profissionais de enfermagem atuantes em assistência direta nas unidades selecionadas.

### Critérios de seleção

Foram incluídas as máscaras N95/PFF2 utilizadas por profissionais de enfermagem durante o turno diurno, em conformidade com os protocolos de reutilização (sete e quinze dias). A escolha desse turno deveu-se ao maior número de procedimentos realizados nesse período. Foram excluídas as máscaras N95/PFF2 utilizadas por profissionais que não seguiram os intervalos de tempo determinados pelos protocolos de reutilização.

### Definição da amostra

 A amostra de conveniência foi subdividida em dois grupos de comparação: um grupo com cinco máscaras N95/PFF2 utilizadas por sete dias e outro com cinco utilizadas por quinze dias, ambos seguindo o protocolo de reutilização estabelecido. É importante destacar que todas as máscaras N95/PFF2 eram da mesma marca e modelo, com tamanho único padrão. Este modelo em particular é caracterizado por suas duas tiras de fixação de material elástico, clipe nasal metálico sem revestimento para ajuste personalizado e pelo *design* dobrável. Além disso, todos os dispositivos possuíam Certificado de Aprovação (CA) emitido pelo Ministério do Trabalho e Emprego (MTE) do Brasil, garantindo que atendem aos rigorosos padrões de segurança e qualidade exigidos pela legislação brasileira. 

### Variáveis do estudo

 As variáveis do estudo foram analisadas utilizando dois instrumentos de coleta de dados, ambos desenvolvidos pelos pesquisadores e baseados nas normativas da *Occupational Safety and Health Administration* dos Estados Unidos da América (EUA) e do Ministério do Trabalho e Emprego do Brasil sobre o uso seguro da máscara N95/PFF2. O primeiro instrumento avaliou a integridade estrutural das máscaras N95/PFF2, considerando o tempo de uso (sete ou quinze dias), a categoria profissional (enfermeiros ou técnicos de enfermagem), e a presença de marcas de identificação pessoal, dobras, furos ou rasgos na superfície, manchas e sujeiras nas partes externa e interna (com respostas de sim ou não para cada item). Caso afirmativo, os tipos de manchas ou sujeiras eram especificados, incluindo vestígios de maquiagem, manchas amareladas ou enegrecidas, café, rabiscos de caneta, entre outros. Para quantificar a extensão da sujidade ou manchas, adotou-se uma estimativa em múltiplos de 25% da área comprometida da máscara N95/PFF2. Essa abordagem incluiu uma avaliação visual, seguida pela classificação das áreas afetadas em categorias conforme sua extensão. Adicionalmente, avaliou-se o estado de conservação das tiras de fixação (íntegras ou esticadas) e do clipe nasal (íntegro, amassado ou com memória de dobras, quebrado, com ranhuras ou outras condições especificadas). 

O segundo instrumento focou nas características morfológicas das fibras das diferentes camadas das máscaras N95/PFF2, incluindo o tempo de uso (sete ou quinze dias), a categoria profissional (enfermeiros ou técnicos de enfermagem), as camadas específicas (camada externa, estrutural, filtrante e interna), as características das fibras (bem preservada, irregular ou altamente irregular), a presença de imperfeições (micro furos, fissuras ou ambos) e detritos. A região escolhida para coleta da amostra foi a área próxima à parte nasal, no lado direito da máscara N95/PFF2, selecionada devido ao maior grau de umidade causado pela respiração do usuário.

### Período, instrumentos utilizados para a coleta das informações e coleta de dados

A coleta de dados foi realizada entre os meses de fevereiro e abril de 2023. Diariamente, no final de cada turno diurno, máscaras N95/PFF2 eram recolhidas conforme um cronograma previamente estabelecido em conjunto com os serviços de saúde. Estas amostras eram acondicionadas em envelopes pardos, etiquetados com informações sobre o local e a data da coleta, marca do dispositivo, período de uso e a categoria profissional do usuário. Posteriormente, eram armazenadas em caixas rígidas que haviam sido higienizadas com água e sabão, em um ambiente arejado. Durante o processo, medidas de segurança foram rigorosamente adotadas, incluindo o uso de avental, luvas de procedimento e máscaras cirúrgicas.

A pesquisadora era responsável pelo transporte das máscaras N95/PFF2, já acondicionadas nas caixas, das unidades de terapias intensiva para o Núcleo de Engenharia da Pontifícia Universidade Católica de Minas Gerias (PUC Minas), em Belo Horizonte, Brasil. No Núcleo, realizava-se a análise da integridade estrutural por meio da inspeção visual à luz natural. Utilizava-se uma lente ótica com ampliação de dez vezes (Tomshin S.A., São Paulo, SP, Brasil) sobre uma bancada desinfetada com álcool 70% para detectar a presença de marcas de identificação, dobras, furos ou rasgos, manchas e sujidade, além de avaliar o estado de conservação dos componentes da máscara N95/PFF2. As marcas de identificação indicam marcações feitas pelo próprio usuário, geralmente com caneta esferográfica, para distinguir seu dispositivo e controlar o tempo de uso. Os resultados foram registrados em formulários individuais para cada amostra.

 Após a coleta e inspeção visual, todas as máscaras N95/PFF2 foram encaminhadas ao Laboratório de Microscopia Avançada da PUC Minas. Neste local, utilizou-se um microscópico eletrônico de varredura (JSM-6610LV, Jeol, Tokio, Japão) para analisar a morfologia das fibras nas diferentes camadas do dispositivo. A manipulação era realizada com luvas e avental não estéreis em uma bancada. Para análise, as amostras foram seccionadas próximo à região do nariz, no lado direito, utilizando uma tesoura previamente desinfetada com álcool 70%. As seções apresentavam dimensões aproximadas de 1,0 por 1,0 cm. Em seguida, as camadas foram separadas e fixadas diretamente em *“stub”* de alumínio com fita adesiva de carbono dupla face. Para tornar as superfícies condutora para a visualização no MEV, uma película de ouro com espessura de quinze nanômetros foi depositada nas amostras utilizando um sistema de revestimento à vácuo de alta pressão (Denton Vaccuum Desk S.A, New Jersey, EUA). As micrografias foram obtidas numa ampliação de 100, 500 e 1000 vezes. Cabe ressaltar que, logo após a chegada ao Núcleo de Engenharia e ao Laboratório de Microscopia, as máscaras N95/PFF2 foram imediatamente submetidas aos testes. Esse procedimento foi adotado para prevenir a influência de quaisquer variáveis ambientais decorrentes de armazenamento em locais inadequados sobre os resultados. 

### Tratamento e análise dos dados

 Utilização do programa *Statistical Package for the Social Sciences* (SPSS), versão 28.0, para análises descritivas e apresentação dos dados em tabelas de distribuição de frequência absoluta e relativa. 

### Aspectos éticos

O estudo seguiu os princípios éticos da Resolução 466/2012 do Conselho Nacional de Saúde, referente à pesquisa envolvendo seres humanos, recebendo aprovação do Comitê de Ética em Pesquisa (CEP) do Brasil, sob o parecer número: 5.824.571 - Certificado de Apresentação de Apreciação Ética (CAAE): 65232922.8.0000.5149. Profissionais de enfermagem foram convidados a participar de maneira voluntária, e lhes foram fornecidas todas as informações pertinentes, assegurando a assinatura individual do Termo de Consentimento Livre e Esclarecido (TCLE). Com intuito de preservar o anonimato, o processo de coleta de dados foi elaborado de forma a excluir qualquer dado que pudesse identificar os indivíduos participantes.

## Resultados

 A integridade das máscaras N95/PFF2 em relação ao protocolo de reutilização (sete e quinze dias) foi avaliada por meio da inspeção visual com auxílio da lente ótica com ampliação de dez vezes da marca Tomshin ^®^ ( Figura 1 ). 

Quanto à estrutura das máscaras N95/PFF2, observou-se a presença de marcas de identificação em 40% (2/5) dos dispositivos que foram reutilizados por sete dias. Essas marcas realizadas pelos técnicos de enfermagem consistiam na escrita das iniciais de seus nomes e da data de recebimento, usando canetas esferográficas na parte externa das máscaras N95/PFF2. O propósito dessas marcações era identificar e controlar o tempo de uso de cada equipamento.

**Figura 1:** - Alterações estruturais em máscaras N95/PFF2 após uso em protocolos de sete (n = 5) e quinze dias (n = 5), em plantão de 12 horas. Belo Horizonte, MG, Brasil, 2023

No que diz respeito à sujidade, após sete dias de uso, 40% (2/5) das máscaras N95/PFF2 apresentavam manchas amareladas ou enegrecidas de origem indeterminada em pelo menos 25% da sua superfície na camada externa, enquanto 60% (3/5) tinham sujidade na camada interna. Para as amostras utilizadas por quinze dias, todas apresentavam sujidade em ambas as camadas, sendo que na face externa as manchas ocupavam uma área menor que 25%, e na camada interna, a maioria 60% (3/5) exibia vestígios de maquiagem em uma área maior que 75%.

Em relação aos componentes das máscaras N95/PFF2 reutilizadas por sete dias, 20% (1/5) das tiras de fixação apresentavam afrouxamento, 20% (1/5) se romperam e 100% (n=5) dos clipes nasais tinham algum tipo dano, sendo que 20% (1/5) estavam amassados ou com memória de dobras, 20% (1/5) além desse dano também apresentaram ranhuras, e 60% (3/5) tinham apenas ranhuras. Em todas as máscaras N95/PFF2 reutilizados por quinze dias, houve afrouxamento das tiras de fixação e comprometimento do clipe nasal, com amassados ou memória de dobras e ranhuras. Além disso, em 40% (2/5) das amostras do protocolo de quinze dias, foi observado desfolhamento entre as camadas na parte interna e presença de curativos de espuma inseridos pelos profissionais de saúde para proteção nasal.

Todas as máscaras N95/PFF2 foram submetidas à microscopia eletrônica de varredura para analisar as alterações morfológicas nas fibras de cada camada, após serem utilizadas nos protocolos de reutilização. Esta análise mostrou que todas as amostras apresentaram modificações morfológicas similares. Diante desta consistência nos achados, foi selecionada uma máscara N95/PFF2 representativa de cada protocolo para ilustração nas figuras do estudo. Em comparação, a máscara N95/PFF2 de controle (não utilizada) exibiu nas micrografias mostraram uma superfície de contorno de fibra bem preservada, sem micro furos, fissuras ou detritos aderidos.

 Ao examinar as camadas externas das amostras de sete dias, observou-se a presença de poros entre as tramas e detritos aderidos às fibras. Nas amostras de quinze dias, foram observados micro furos, fios de fibras soltos, além de detritos aderidos às fibras e poros entre as tramas ( Figura 2 ). 

**Figura 2:** - Microscopia eletrônica de varredura da camada externa da máscara N95/PFF2 controle e das amostras nos protocolos de reuso (sete e quinze dias) durante plantões de 12 horas, com ampliação de 100x, 500x e 1000x. Belo Horizonte, MG, Brasil, 2023

As imagens microscópicas 2(A-C) da camada externa da máscara N95/PFF2 de controle, revelaram fibras bem preservadas. Nas imagens 2(D-F), referentes às amostras de sete dias, observa-se a presença de poros entre as tramas em 2(D) e 2(F) e detritos em 2(E). As imagens 2(G-I), correspondentes às amostras de quinze dias, foram identificados micro furos e fibras soltas em 2(G), detritos em 2(H) e 2(I), e poros entre as tramas em 2(I).

 Na análise das camadas estruturais das máscaras N95/PFF2 após os protocolos de reutilização de sete dias, foram observadas tramas “emaranhadas”, rompimento das fibras e detritos aderidos às mesmas. Nas amostras reutilizadas por quinze dias, além das tramas “emaranhadas” e detritos aderidos, também foram identificados desgastes nas fibras ( Figura 3 ). 

 As imagens microscópicas 3(A-C) da máscara N95/PFF2 de controle mostraram fibras bem preservadas. A imagem 3(D) das amostras de sete dias revelou tramas “emaranhadas”, a imagem 3(E) evidenciou rompimento das fibras e detritos, enquanto a imagem 3(F) mostrou apenas detritos. As imagens 3(G-I) das amostras de quinze dias revelaram tramas “emaranhadas” em 3(G), detritos em 3(H) e detritos com desgaste das fibras em 3(I). Em relação às camadas filtrantes das máscaras N95/PFF2 submetidas ao protocolo de reutilização de sete e quinze dias, foi observado a presença de tramas “emaranhadas” e rompimento das fibras. Nas amostras de quinze dias, além de tramas “emaranhadas”, houve a contestação de poros maiores entre as tramas ( Figura 4 ). 

**Figura 3:** - Microscopia eletrônica de varredura da camada estrutural da máscara N95/PFF2 controle e das amostras nos protocolos de reuso (sete e quinze dias) durante plantões de 12 horas, com ampliação de 100x, 500x e 1000x. Belo Horizonte, MG, Brasil, 2023

**Figura 4:** - Microscopia eletrônica de varredura da camada filtrante da máscara N95/PFF2 controle e das amostras nos protocolos de reuso (sete e quinze dias) durante plantões de 12 horas, com ampliação de 100x, 500x e 1000x. Belo Horizonte, MG, Brasil, 2023

As imagens 4(A-C) da máscara N95/PFF2 de controle mostraram fibras bem preservadas. As imagens 4(D-F) das amostras de sete dias revelaram tramas “emaranhadas”. As imagens 4(G-H) das amostras de quinze dias também revelaram tramas “emaranhadas”, enquanto a imagem 4(I) revelou poros entre as tramas.

 Ao analisar as camadas internas das máscaras N95/PFF2 utilizadas nos protocolos de sete dias, foram identificados micro furos, detritos aderidos às fibras e poros entre as tramas. Nas amostras reutilizadas nos protocolos de quinze dias, revelou além desses danos, a presença de fios de fibras soltos ( Figura 5 ). 

**Figura 5:** - Microscopia eletrônica de varredura da camada interna da máscara N95/PFF2 controle e das amostras nos protocolos de reuso (sete e quinze dias) durante plantões de 12 horas, com ampliação de 100x, 500x e 1000x. Belo Horizonte, MG, Brasil, 2023

As imagens 5(A-C) da máscara N95/PFF2 de controle mostraram fibras bem preservadas. A imagem 5(D) das amostras de sete dias revelou a presença de micro furos, detritos e poros entre as tramas, enquanto as imagens 5(E) e 5(F) revelaram poros entre as tramas. A imagem 5(G) das amostras de quinze dias também revelou poros entre as tramas, e as imagens 5(H) e 5(I) mostraram detritos e poros entre as tramas, bem como fios soltos de fibras.

## Discussão

Na análise de máscaras N95/PFF2 submetidas a protocolos de reutilização por períodos de sete e quinze dias, verificou-se alterações substanciais atribuíveis a esta prática, afetando tanto a integridade estrutural quanto a morfologia das fibras. Este fato sugere um risco aumentado para a segurança dos profissionais de saúde, ressaltando a necessidade de uma abordagem cautelosa e criteriosa no reuso desses equipamentos de proteção.

 Essas descobertas são de grande relevância, dado que a implementação desses protocolos de reutilização foi essencial para atender à elevada demanda mundial durante a pandemia da COVID-19, bem como em epidemias anteriores, como a influenza H1N1 em 2009, Síndrome Respiratória do Oriente Médio em 2012 ^(^ ^18^ ^-^ ^21^ ^)^ . Além disso, esta estratégia ainda é adotada como uma política de redução de custos financeiros em diversas instituições hospitalares ao redor do mundo, especialmente em países que enfrentam impactos desproporcionais da COVID-19, como Brasil, Índia e China, com altos números de casos e mortalidade, além de uma alta incidência de outras doenças respiratórias, como tuberculose ^(^ ^22^ ^-^ ^24^ ^)^ . 

 Ao avaliar a integridade das máscaras N95/PFF2 reutilizadas por sete e quinze dias, identificaram-se diversos sinais de desgaste nas tiras de fixação e degradações nos clipes nasais por meio da inspeção visual. Esses danos corroboram com as descobertas de estudos realizados tanto em ambientes clínicos quanto em laboratoriais, evidenciando a fragilidade desses equipamentos sob condições de reuso ^(^ ^9^ ^,^ ^25^ ^)^ . Uma pesquisa clínica realizada em um hospital terciário na Índia, durante a pandemia da COVID-19, indicou que 9% dos profissionais de saúde relataram o afrouxamento das tiras de fixação, geralmente após cinco reuso em turnos de oitos horas, como uma das principais justificativas para o descarte precoce das máscaras N95/PFF2 ^(^ ^25^ ^)^ . Em um contexto laboratorial, simulando um turno de dez horas, constatou-se que a ruptura de cinco clipes nasais e de oito itens de fixação, ocorridas ao longo de 20 episódios de paramentação e desparamentação, resultou na falha nos testes de vedação por todos os participantes que utilizavam tais dispositivos ^(^ ^9^ ^)^ . 

 A degradação dos componentes das máscaras N95/PFF2 pode ser exacerbada pela intensa rotina dos profissionais de saúde, que frequentemente necessitam paramentar-se e desparamentar-se várias vezes durante a jornada de trabalho, especialmente para intervalos essenciais como hidratação, alimentação e, até mesmo, descanso da face ^(^ ^9^ ^,^ ^15^ ^)^ . As pesquisas realizadas em serviços de saúde mostram uma variação na frequência de paramentações, variando de até cinco ocorrências em turnos de dez horas e uma média de quarenta em jornadas de doze horas ^(^ ^26^ ^-^ ^27^ ^)^ . Portanto, é vital que os usuários realizem inspeções visuais rigorosas e verifiquem a vedação das máscaras N95/PFF2 imediatamente antes de cada uso para assegurar a manutenção da integridade desses dispositivos e, consequentemente, garantir proteção eficaz contra agentes infecciosos transmitidos pelo ar ^(^ ^4^ ^-^ ^5^ ^,^ ^28^ ^)^ . Entretanto, um estudo recente revelou que 57% dos enfermeiros não efetuam essas verificações, destacando a urgente necessidade de treinamentos frequentes e maior conscientização sobre a essencialidade desses procedimentos para o uso correto e seguro do equipamento de proteção individual ^(^ ^4^ ^,^ ^29^ ^)^ . 

 Outro ponto crítico identificado no presente estudo foi a incidência alarmante de sujidade nas máscaras N95/PFF2, uma realidade que contraria as diretrizes sanitárias, as quais recomendam a substituição imediata de dispositivos que estejam sujos, úmidos ou danificados ^(^ ^2^ ^,^ ^4^ ^)^ . Esta observação é corroborada por uma pesquisa realizada em ambiente hospitalar, onde a inspeção visual revelou a presença de manchas ou sujeira em todos máscaras N95/PFF2 utilizadas por auxiliares de enfermagem após cinco de uso, sendo os resíduos de cosméticos os mais comuns ^(^ ^10^ ^)^ . Esses achados sublinham a necessidade crítica de aderir às práticas recomendadas pelos órgãos de saúde, tanto em termos de inspeção quanto a manutenção dos equipamentos livres de contaminação e sujeira, além de íntegros para garantir a proteção efetiva dos profissionais de saúde. Todavia, ressalta-se que o uso de maquiagem intensifica a deterioração das fibras, o que por sua vez, propicia a diminuição da sua eficácia ^(^ ^4^ ^-^ ^5^ ^)^ . 

 As instituições hospitalares participantes do estudo, em conformidade com as orientações estabelecidas pelos órgãos de saúde ANVISA e CDC, providenciaram locais próprios, limpos e arejados para o armazenamento das máscaras N95/PFF2 ^(^ ^2^ ^,^ ^4^ ^)^ . Além disso, promoveram o uso de envelopes de papel, que facilitam a circulação de ar e permitem a identificação pessoal, evitando a marcações diretas nos dispositivos. Essas práticas visam preservar a integridade estrutural do EPI e reduzir o risco de trocas inadvertidas entre os usuários. No entanto, após os protocolos de sete e quinze dias, observou-se dobras e marcas feitas por profissional com canetas esferográficas nas amostras, sugerindo um possível acondicionamento impróprio das máscaras N95/PFF2 em bolsos de vestimentas ou em itens pessoais, como bolsas. Essa condição também é constatada em outros estudos, chamando a atenção para a crucialidade de implementar ações de conscientização para desencorajar a guarda inadequada desses dispositivos ^(^ ^25^ ^,^ ^27^ ^)^ . 

 Outro aspecto que merece destaque no contexto do protocolo de reutilização das máscaras N95/PFF2 é a ocorrência de eventos adversos, incluindo lesões por pressão na ponte nasal devido ao atrito e cisalhamento causado pelo deslocamento do dispositivo ^(^ ^30^ ^-^ ^31^ ^)^ . Na prática clínica, é comum que os profissionais de saúde utilizem adesivos de silicone ou espuma na parte interna das máscaras N95/PFF2 para proteger a pele contra danos e proporcionar conforto na região, principalmente no nariz, devido à estrutura metálica do clipe nasal ^(^ ^32^ ^-^ ^33^ ^)^ . Essas estratégias foram observadas em duas amostras reutilizadas em protocolos de quinze dias neste estudo. Contudo, esses curativos de espuma podem comprometer o ajuste desses dispositivos, em virtude da possibilidade de folga entre a pele e o equipamento de proteção respiratória ^(^ ^32^ ^)^ . 

 As máscaras N95/PFF2, certificadas pelo *National Institute for Occupational Safety and Health* (NIOSH), são constituídas de microfibras sintéticas de polipropileno, distribuídas em quatro camadas distintas: externa, estrutural, filtrante e interna ^(^ ^28^ ^,^ ^34^ ^)^ . As camadas externa e interna são especificamente projetadas para proteção contra a umidade, com fibras de diâmetros entre 20 e 25 mícrons ^(^ ^34^ ^)^ . Por outro lado, o meio filtrante é composto por fibras menores, de dois a cinco mícrons, tratadas eletrostaticamente para reter no mínimo 95% das partículas aéreas de até 0,3 µm ^(^ ^34^ ^)^ . 

A partir do sétimo dia de uso das máscaras N95/PFF2, as análises por microscopia eletrônica de varredura revelaram uma tendência ao aumento dos poros na maioria das camadas e a presença de tramas “emaranhadas” nas camadas estrutural e filtrante, possivelmente decorrentes do desprendimento das fibras, criando espaços maiores entre elas. Essas alterações se intensificaram no protocolo de quinze dias de uso, destacando a relação direta entre o tempo de reuso e a magnitude dos resultados observados.

 No contexto da saúde ocupacional, essas mudanças morfológicas nas fibras dos dispositivos levantam preocupações, pois aumentam substancialmente a possibilidade de penetração de sangue, secreções respiratórias ou nasais e outros fluidos corporais através das camadas das máscaras N95/PFF2. Esse cenário é especialmente crítico em ambientes como unidades de terapia intensiva, onde procedimentos que geram aerossóis são rotineiros e apresentam alto risco de transmissão de vírus respiratórios ^(^ ^35^ ^-^ ^36^ ^)^ . 

 Além disso, estudo adicional enfatiza que máscaras N95/PFF2 com poros menores, mais próximas às suas características originais, oferecem uma eficiência de filtração superior ^(^ ^37^ ^)^ Isso significa que maiores espaços entre as tramas favorecem a penetração de um maior número de partículas aerossolizada, ampliando a vulnerabilidade desses profissionais ^(^ ^37^ ^-^ ^38^ ^)^ . 

Micrografias das máscaras N95/PFF2 também evidenciaram a presença de micro furos e o rompimento das fibras, possivelmente decorrentes do esgarçamento, o que leva à ampliação dos espaços de retenção de partículas e facilita acumulação de detritos entre às fibras. Elementos observados durante a inspeção visual, como sujidade, dobras e marcas de identificação pessoal nas amostras podem ter influenciado essas alterações morfológicas. Portanto, é crucial estabelecer protocolos adequados e limites de reuso desses dispositivos para assegurar a proteção necessária aos profissionais de saúde.

 Este estudo, apesar de seus *insights* valiosos, apresenta algumas limitações que devem ser consideradas ao interpretar seus achados. Inicialmente, a utilização de uma amostra reduzida pode restringir a generalização das conclusões. Além disso, a análise focada exclusivamente em um tipo específico de máscara N95/PFF2, de uma determinada marca e modelo, possibilita restringir a aplicabilidade dos resultados a esse tipo em particular. Por último, a ausência de controle direto dos pesquisadores sobre o armazenamento das máscaras N95/PFF2, apesar dos protocolos e infraestrutura fornecidos pelas instituições, podem ter introduzido uma variável não monitorada, com potencial impacto nos resultados. 

Apesar dessas limitações, o uso de técnicas de inspeção visual e a microscopia eletrônica de varredura possibilitaram identificar uma lacuna expressiva em relação ao impacto dos protocolos de reutilização das máscaras N95/PFF2, especialmente em relação à integridade e à funcionalidade das características inerentes a cada camada dos dispositivos após serem submetidos aos protocolos de reutilização por períodos de sete e quinze dias. Portanto, a reutilização desses equipamentos, apesar de ser uma recomendação emanada de órgão sanitários internacionais e nacionais, configura um risco tangível à segurança dos profissionais de saúde.

As implicações desse estudo para o avanço do conhecimento científico na área da saúde e enfermagem são notáveis. Ao identificar alterações na integridade estrutural e morfológica das máscaras N95/PFF2 após a reutilização, destaca-se a importância de reavaliar os protocolos existentes e desenvolver diretrizes mais eficazes. Dessa forma, essa pesquisa incentiva a realização de testes adicionais para garantir a proteção adequada dos profissionais de saúde.

## Conclusão

Protocolos de reutilização de sete e quinze dias influenciaram potencialmente a ocorrência de alterações morfológicas nas fibras das camadas das máscaras N95/PFF2, além da degradação tanto da integridade estrutural quanto dos componentes de fixação. Esses achados sugerem que a reutilização desses dispositivos respiratórios na prática clínica acarreta uma redução expressiva da eficácia do equipamento no que tange a integridade e funcionalidade das características de vedação e filtração, comprometendo, portanto, a segurança dos profissionais de saúde.
